# PD-1/PD-L1 Based Combinational Cancer Therapy: Icing on the Cake

**DOI:** 10.3389/fphar.2020.00722

**Published:** 2020-05-15

**Authors:** Jian-ye Zhang, Yan-yan Yan, Jia-jun Li, Rameshwar Adhikari, Li-wu Fu

**Affiliations:** ^1^State Key Laboratory of Oncology in Southern China, Cancer Center, Sun Yat-Sen University, Guangzhou, China; ^2^Key Laboratory of Molecular Target & Clinical Pharmacology, School of Pharmaceutical Sciences & the Fifth Affiliated Hospital, Guangzhou Medical University, Guangzhou, China; ^3^Institute of Respiratory and Occupational Diseases, Collaborative Innovation Center for Cancer, Medical College, Shanxi Datong University, Datong, China; ^4^Research Centre for Applied Science and Technology, Tribhuvan University, Kirtipur, Nepal

**Keywords:** cancer, PD-1, PD-L1, immunotherapy, combinational therapy

## Abstract

Cancer has been a major global health problem due to its high morbidity and mortality. While many chemotherapy agents have been studied and applied in clinical trials or in clinic, their application is limited due to its toxic side effects and poor tolerability. Monoclonal antibodies specific to the PD-1 and PD-L1 immune checkpoints have been approved for the treatment of various tumors. However, the application of PD-1/PD-L1 inhibitors remains suboptimal and thus another strategy comes in to our sight involving the combination of checkpoint inhibitors with other agents, enhancing the therapeutic efficacy. Various novel promising approaches are now in clinical trials, just as icing on the cake. This review summarizes relevant investigations on combinatorial therapeutics based on PD-1/PD-L1 inhibition.

## Introduction

Cancer has become one of the major problems threatening human health based on its high rates of morbidity and mortality (Huang and Fu, 2015; Zhang et al., 2016; Zhang et al., 2017; Huang et al., 2020). Chemotherapeutic drugs play a major role in cancer treatment (Shi et al., 2011; Lin et al., 2017a; Lin et al., 2017b; Jiang et al., 2019). It is undeniable that these treatments are effective at present, but they also destroy the physiological state of normal cells while killing tumor cells, resulting in irreversible damage and therefore poor patient tolerability (Shi et al., 2007; Kathawala et al., 2015; Siegel et al., 2018; Liu et al., 2019). Recently, cancer immunotherapy has been on the rise. It has been shown that immunotherapy has achieved excellent therapeutic efficacy in a variety of tumors, including melanoma, non-small cell lung cancer, renal cell carcinoma, colorectal cancer, as well as breast cancer (Hanahan and Weinberg, 2011; Siegel et al., 2017; Sanmamed and Chen, 2018; Yu et al., 2019). Antibodies specifically against programmed death-1 (PD-1), programmed death-ligand 1 (PD-L1), and cytotoxic T lymphocyte antigen 4 (CTLA-4) (e.g., ipilimumab, tremelimumab) are regarded as recent breakthroughs in cancer immunotherapy (Quezada and Peggs, 2013; Herbst et al., 2014; Turajlic et al., 2018; Rahimi Kalateh Shah Mohammad et al., 2020).

## PD-1/PD-L1 Overview

**Figure 1:**
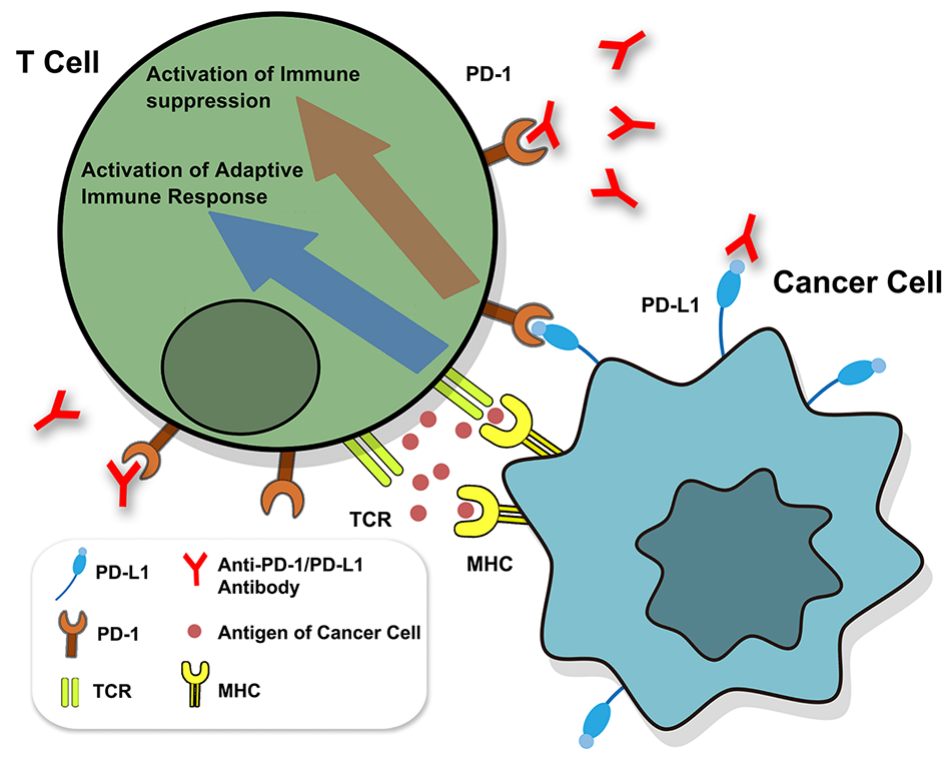
Identification of PD-1/PD-L1. The binding of TCR and MHC activates adaptive immune response. The binding of PD1 and PD-L1 can prevent the signaling transduction of T cells to inhibit the immune response, while anti-PD-1/PD-L1 antibody can reverse the inhibition. TCR, T cell receptor.

PD-1 pertains to a suppressive T-cell receptor that is generally expressed by activated T cells, and antigen-specific T cells, which are chronically exposed to various antigens (Day et al., 2006; Tian et al., 2019; Wang and Wei, 2019). PD-1 is highly selective for immune-inhibitory signals that are mediated by programmed death-ligand 1 (PD-L1, B7-H1), which is generated by malignant cells, myeloid-derived suppressor cells (MDSCs), and leukocytes (Iwai et al., 2002; Blank et al., 2004; Von Knethen and Brüne, 2019). Cancer cells escape immune responses by overexpressing PD-L1 (**Figure 1**) (Okazaki and Honjo, 2007; Markham, 2016; Cao et al., 2019). The immune system is activated by diseases, whereas PD-L1 inhibits the immune system by preventing foreign antigen-specific T cells from accumulating and reducing antigen-specific CD8^+^ T cell proliferation (Trautmann et al., 2006; Sanmamed and Chen, 2018). The inhibitory effect of therapeutic antibodies on PD-1/PD-L1 is expected to be highly specific to tumor antigen-specific T cells and exhibits lower specificity for auto-reactive T cells (Sznol and Chen, 2013; Homet Moreno et al., 2015). It has been recently confirmed that PD-1/PD-L1 treatment can regulate T-cell activation, including the disruption of suppression of T cell receptor (TCR) activation that is caused by PI3K/Akt/Ras-MEK/ERK, as well as the negative feedback loop involving the cell cycle, thereby leading to apoptosis (Day et al., 2006; Butte et al., 2007; Quigley et al., 2010; Markham, 2016; Kamta et al., 2017; Li X. et al., 2019).

## Drugs Targeting PD-1/PD-L1

Until now, six PD-1/PD-L1 targeted drugs have been listed in dozens of countries in Europe and United States, which are made up of three PD-1 antibodies and three PD-L1 antibodies (Sanmamed and Chen, 2018). See **Table 1** for details. In addition, four innovative anti-PD-1/PD-L1 mAbs have been on the Chinese market, including toripalimab, sintilimab, camrelizumab, and tislelizumab.

**Table 1:** Six PD-1/PD-L1 targeted drugs.

Abbreviation	O drug	K drug	T drug	I drug	B drug	L drug
**Trade name**	Opdivo	Keytruda	Tecentriq	Imfinzi	Bavencio	Libtayo
**Common name**	Nivolumab	Pembrolizumab	Atezolizumab	Durvalumab	Avelumab	Cemiplimab
**Manufacturer**	Bristol-Myers Squibb, USA	Merck, USA	Genentech, USA	AstraZeneca, UK	Merck, USA	Regeneron Pharmaceuticals Inc
**Target**	PD-1	PD-1	PD-L1	PD-L1	PD-L1	PD-1
**Indication**	Melanoma, metastatic squamous NSCLC, etc.	Melanoma, NSCLC, renal cell carcinoma, head and neck squamous cell carcinoma, etc.	Urothelial carcinoma	NSCLC, urothelial carcinoma	Merkel cell carcinoma, urothelium carcinoma	metastatic cutaneous squamous cell carcinoma (CSCC) or locally advanced CSCC who are not candidates for curative surgery or curative radiation.
**Approval year**	2014	2014	2016	2017	2017	2018
**Time to market**	2018	2018	–	–	–	

## Anti-PD-1/PD-L1 Drugs Based Combinational Therapy

### Nivolumab Based Combinational Therapy

#### Preclinical Study

Synergistic antitumor activity in mouse MC38 and CT26 colorectal tumor models was observed with concurrent, but not sequential CTLA-4 and PD-1 blockade. Significant antitumor activity was maintained using a fixed dose of anti-CTLA-4 antibody with decreasing doses of anti-PD-1 antibody in the MC38 model. Immunohistochemical and flow cytometric analyses confirmed that CD3^+^ T cells accumulated at the tumor margin and infiltrated the tumor mass in response to the combination therapy, resulting in favorable effector and regulatory T-cell ratios, increased pro-inflammatory cytokine secretion, and activation of tumor-specific T cells. Similarly, *in vitro* studies with combined ipilimumab and nivolumab showed enhanced cytokine secretion in superantigen stimulation of human peripheral blood lymphocytes and in mixed lymphocyte response assays. In a cynomolgus macaque toxicology study, dose-dependent immune-related gastrointestinal inflammation was observed with the combination therapy, which had not been observed in previous single agent cynomolgus studies. Together, these *in vitro* assays and *in vivo* models comprise a preclinical strategy for the identification and development of highly effective antitumor combination immunotherapies (Selby et al., 2016).

#### Melanoma

The first clinical trial of combinational treatment of PD-1 plus CTLA-4 inhibitors was reported in 2013 (Wolchok et al., 2013). Here, 53 melanoma patients were treated with nivolumab + ipilimumab, whereas 33 patients received nivolumab alone. Results showed that the efficacy of the combinatorial treatment was superior to ipilimumab or nivolumab alone as earlier reported. In the combinatorial treatment group, the 2-year survival was 79%, and the objective response rate (ORR) was 42%. Responding patients showed an 80% tumor reduction, and 17% of the patients had a complete response (Pico De Coaña et al., 2015). Nivolumab monotherapy and combination with ipilimumab increase proportions of patients achieving a response and survival, versus ipilimumab in patients with metastatic melanoma. In 2015, the United States Food and Drug Administration (USFDA) approved ipilimumab + nivolumab for the treatment of metastatic or unresectable melanoma (Swart et al., 2016).

In a double-blind study involving 142 patients with metastatic melanoma who had not previously received treatment, the ORR and the progression-free survival (PFS) were significantly greater with nivolumab combined with ipilimumab, than that with ipilimumab monotherapy. Combination therapy had an acceptable safety profile (Postow et al., 2015). In a phase 1 dose-escalation study, combined inhibition of T-cell checkpoint pathways by nivolumab and ipilimumab was associated with a high ORR, including complete responses, among patients with advanced melanoma. In the advanced melanoma (CheckMate 067), the phase 2 trial (at 2 years of follow-up) revealed that the combination of first-line nivolumab plus ipilimumab might lead to improved outcomes, compared with first-line ipilimumab alone (Hodi et al., 2016). Nivolumab combined with ipilimumab resulted in longer progression-free survival and a higher ORR than ipilimumab alone in a phase 3 trial involving patients with advanced melanoma. In the advanced melanoma patients, significantly longer overall survival (OS) occurred with combination therapy of nivolumab plus ipilimumab or nivolumab alone, than with ipilimumab alone (Wolchok et al., 2017). The following phase 3 trial (at 4 years of follow-up) showed that a durable, sustained survival benefit can be achieved with first-line nivolumab plus ipilimumab or nivolumab alone in the advanced melanoma patients (Hodi et al., 2018). Among patients with advanced melanoma, sustained long-term OS at 5 years was observed in a greater percentage of patients who received nivolumab plus ipilimumab or nivolumab alone, than monotherapy of ipilimumab. In addition, no patients who received regimens containing nivolumab got apparent loss of quality of life. These results suggest encouraging survival outcomes with immunotherapy in this population of patients (Larkin et al., 2019).

In addition, a multicenter open-label randomized phase 2 trial (NCT02374242) was done and revealed nivolumab combined with ipilimumab and nivolumab monotherapy were active in melanoma brain metastases. A high proportion of patients achieved an intracranial response with the combination. Thus, nivolumab combined with ipilimumab should be considered as a first-line therapy for patients with asymptomatic untreated brain metastases (Long et al., 2018).

The above are some evidence that PD-1 and CTLA-4 are efficacious *via* dependent immune pathways. The simultaneous inhibition of both pathways can induce synergistic effects.

#### NSCLC and SCLC

A single-center phase Ib study investigated the tolerability, safety, and pharmacokinetics of nivolumab combined with standard chemotherapy in patients with advanced non-small-cell lung cancer (NSCLC). Results indicated that combination of nivolumab 10 mg/kg and chemotherapy showed an acceptable toxicity profile and encouraging antitumor activity in patients with advanced NSCLC (Kanda et al., 2016). In three academic hospitals in the USA, an open-label, non-randomized, phase Ib clinical trial was conducted with patients with ages ≥18 years. These individuals were previously treated histologically or confirmed cytologically to be at stage IIIB or IV NSCLC. From January 2016 to June 2017, 21 patients received ALT-803 (an IL-15 superagonist) plus nivolumab at four dose levels. The results showed that the ALT-803 + nivolumab is safe in the outpatient setting, using a dose of ALT-803 at 20 μg/kg that was administered subcutaneously once per week plus nivolumab administered intravenously at 240 mg every 2 weeks. This is the first report on using IL-15 in the treatment of patients with NSCLC, the potential of ALT-803 + nivolumab (Wrangle et al., 2018). In addition, Oshima Y, et al. found a higher proportion of reports about Interstitial Pneumonitis (IP) for nivolumab in combination with EGFR-TKI, than treatment with either drug alone, including concomitant and sequential use, and careful monitoring for IP is recommended (Oshima et al., 2018; Li D. et al., 2019).

Hellmann MD, et al. indicated that in SCLC patients, nivolumab plus ipilimumab appeared to provide a greater clinical benefit than nivolumab monotherapy in the high tumor mutational burden tertile (Hellmann et al., 2018).

#### Metastatic Sarcoma

Patients with metastatic sarcoma have limited treatment options. In the two open-label, non-comparative, randomized, phase 2 trials (NCT02500797), the activity and safety of nivolumab alone or in combination with ipilimumab in patients with locally advanced, unresectable, or metastatic sarcoma were investigated. The results indicated nivolumab combined with ipilimumab demonstrated promising efficacy in certain sarcoma subtypes, with a manageable safety profile comparable to current available treatment options. The combination therapy met its predefined primary study endpoint; further evaluation of nivolumab plus ipilimumab in a randomized study is warranted (D'angelo et al., 2018).

#### Renal-Cell Carcinoma

Purpose combination treatment with immune checkpoint inhibitors has shown enhanced antitumor activity. The open-label, parallel-cohort, dose-escalation, phase I CheckMate 016 study evaluated the efficacy and safety of nivolumab plus ipilimumab, and nivolumab plus a tyrosine kinase inhibitor in metastatic renal cell carcinoma (mRCC). This investigation showed that nivolumab plus ipilimumab therapy demonstrated manageable safety, notable antitumor activity, and durable responses with promising OS in patients with mRCC (Hammers et al., 2017).

OS and ORR were significantly higher with nivolumab plus ipilimumab than with sunitinib among intermediate- and poor-risk patients with previously untreated advanced renal-cell carcinoma. Further study showed that treatment-related adverse events, grade 3 or 4 events, and treatment-related adverse events leading to discontinuation were lower in the nivolumab-plus-ipilimumab group than in the sunitinib group (Motzer et al., 2018).

#### Lymphoma

In the phase 1/2 study, brentuximab vedotin (BV) and nivolumab administered in combination was an active and well-tolerated first salvage regimen, potentially providing patients with R/R HL an alternative to traditional chemotherapy (Clinical Trials: NCT02572167) (Herrera et al., 2018).

Combining local irradiation with anti-PD-1 checkpoint blockade treatment is feasible and synergistic in refractory Hodgkin's lymphoma. Correlative studies also suggest that the expression of PD-L1, DNA damage response, and mutational tumor burden can be used as potential biomarkers for treatment response (Qin et al., 2018).

The combination of ibrutinib and nivolumab had an acceptable safety profile and preliminary activity was similar to that reported with single-agent ibrutinib in chronic lymphocytic leukemia or small lymphocytic lymphoma, follicular lymphoma, and diffuse large B-cell lymphoma (Clinical Trials: NCT02329847) (Younes et al., 2019).

#### Colorectal Cancer

The clinical trial CheckMate-142 evaluated the efficacy and safety of nivolumab + ipilimumab in 119 patients with microsatellite instability-high (MSI-H)/DNA mismatch repair-deficient (dMMR) metastatic colorectal cancer (mCRC). The patients received a combination of 3 mg/kg nivolumab and 1 mg/kg ipilimumab at 3-week intervals (for a total of four doses), followed by nivolumab 3 mg/kg at 2-week intervals (Gourd, 2018; Overman et al., 2018). Approximately 76% of patients earlier received two or more systemic treatments. The nivolumab + ipilimumab regimen showed acceptable tolerability, high response rate, and significantly higher PFS and OS at 12-month follow-up. Nivolumab + ipilimumab was thus considered as a potential novel treatment option for patients with dMMR/MSI-H mCRC (Sznol, 2014; Gourd, 2018).

The details for clinical trials of nivolumab based combinational therapy were summarized in **Table 2**.

**Table 2:** Nivolumab based combinational therapy.

Cancer type	Treatment	Dose schedule	Efficacy	Adverse rate	Notes	References
Melanoma	Nivolumab ± ipilimumab	N + I q3w × 4 doses, followed by N q3w × 4 doses, continued q12w for up to 8 doses Escalating doses of N: 0.3, 1, 3, 10 mg/kg; of I: 1, 3, 10 mg/kg	All: 40% ORR Acceptable level of AEs (1 mg/kg N + 3 mg/kg I): 53% ORR	53% Grade 3/4 AEs	NCT01024231 Patients with a diagnosis of measurable, unresectable, stage III or IV melanoma;	(Wolchok et al., 2013)
		N q2w for up to 48 doses (previously treated with ipilimumab) Escalating doses of N: 1, 3 mg/kg	20% ORR 73.4% OS			
		1 mg/kg N + 3 mg/kg I q3w for 4 doses, followed by 3 mg/kg N q2w	59% ORR 73.4% OS	92% AEs	NCT01927419 CheckMate 069 Patients with unresectable stage III or IV melanoma	(Hodi et al., 2016)
		3 mg/kg I q3w × 4 doses	11% ORR 63.8% OS	94% AEs		
		1 mg/kg N + 3 mg/kg I q3w × 4 doses, followed by 3 mg/kg N q2w	58% ORR 58% OS	59% Grade 3/4 AEs	NCT01844505 CheckMate 067 Patients with stage III (unresectable) or stage IV melanoma	(Wolchok et al., 2017)
		3 mg/kg N q2w × 4 doses	44% ORR 52% OS	21% Grade 3/4 AEs		
		3 mg/kg I q3w × 4 doses	19% ORR 34% OS	28% Grade 3/4 AEs		
		1 mg/kg N + 3 mg/kg I q3w × 4 doses, followed by 3 mg/kg N q2w	58% ORR	59% Grade 3/4 AEs	NCT01844505 Patients with unresectable or stage III or stage IV melanoma,	(Hodi et al., 2018)
		3 mg/kg N q2w ×4 doses	45% ORR	22% Grade 3/4 AEs		
		3 mg/kg I q3w × 4 doses	19% ORR	28% Grade 3/4 AEs		
		1 mg/kg N + 3 mg/kg I q3w × 4 doses, followed by 3 mg/kg N q2w	58% ORR 22% CR	59% Grade 3/4 AEs	NCT01844505 CheckMate 067	(Larkin et al., 2019)
		3 mg/kg N q2w	45% ORR 19% CR	23% Grade 3/4 AEs		
		3 mg/kg I every 3 weeks × 4 doses	19% ORR 6% CR	28% Grade 3/4 AEs		
		1 mg/kg N + 3 mg/kg I q3w × 4 doses, then 3 mg/kg N q2w	46% ORR 78% OS	97% TRAEs 54% Grade 3/4 AEs	NCT02374242 Patients with melanoma brain metastases	(Long et al., 2018)
		3 mg/kg N q2w	20% ORR 68% OS	68% TRAEs 16% Grade 3/4 AEs		
		3 mg/kg N q2w (local therapy failed, neurological symptoms, or leptomeningeal disease)	6% ORR 44% OS	50% TRAEs 13% Grade 3/4 AEs		
Lung cancer	Nivoluma + standard chemotherapy	10 mg/kg N (D1) + 1250 mg/m^2^ gemcitabine (D1 and 8) + 80 mg/m^2^ cisplatin (D1), q3w for up to four cycles, followed by 10 mg/kg N (D1) q3w	50% ORR 6.28 months mPFS	66.7% Grade 3/4 AEs	JapicCTI-132071 Patients with stage IIIB (without indication for definitive radiotherapy) stage IV, or recurrent NSCLC	(Kanda et al., 2016)
		10 mg/kg N (D1) + 500 mg/m^2^ pemetrexed (D1) + 75 mg/m^2^ cisplatin (D1) q3w for up to four cycles, followed by 10 mg/kg N (D1) + 500 mg/m^2^ pemetrexed (D1) q3w	50% ORR 9.63 months mPFS	66.7% Grade 3/4 AEs		
		10 mg/kg N (D1) + 200 mg/m^2^ paclitaxel (D1) + 6 mg/ml/min (AUC) carboplatin (D1) + 15 mg/kg bevacizumab (D1) q3w for up to six cycles, followed by 10 mg/kg N (D1) + 15 mg/kg bevacizumab (D1) q3w	100% ORR None mPFS	100% Grade 3/4 AEs		
		10 mg/kg N (D1) + 75 mg/m^2^ docetaxel (D1)	16.7% ORR 3.15 months mPFS	100% Grade 3/4 AEs		
	ALT-803 + nivolumab	3 mg/kg N q2w + ALT-803 q1w × four cycles Escalating dose of ALT-803: 6, 10, 15, or 20 μg/kg	ORR 29% 17.4 months mPFS	–	NCT02523469 Patients with IIIB or IV NSCLC (or recurrent disease following previous radiotherapy or surgical resection)	(Wrangle et al., 2018)
	Nivolumab ± ipilimumab	1 mg/kg N + 3 mg/kg I q3w for four cycles, followed by 3 mg/kg N q2w	46.2% ORR	–	NCT01928394 CheckMate 032 Patients with limited- or extensive-stage SCLC with progression after at least one platinum-based chemotherapy regimen	(Hellmann et al., 2018)
		3 mg/kg N q2w	21.3% ORR	–		
Metastatic sarcoma	Nivolumab ± ipilimumab	3 mg/kg N + 1 mg/kg I q3w for 4 doses, followed by 3 mg/kg N q2w for up to 2 years	16% ORR 4.1 months mPFS	26% Serious TRAEs	NCT02500797 Patients with bone or soft tissue sarcoma, locally advanced, unresectable, or metastatic sarcoma	(D'angelo et al., 2018)
		3 mg/kg N q2w, followed by 3 mg/kg N q2w for up to 2 years	5% ORR 1.7 months mPFS	19% Serious TRAEs		
Renal-cell carcinoma	Nivolumab + ipilimumab	3 mg/kg N + 1mg/kg I q3w × 4 doses, followed by 3 mg/kg N q2w	40.4% ORR 67.3% OS	38.3% Grade 3/4 TRAEs	CheckMate 016 Patients with advanced RCC or mRCC with a clear-cell component	(Hammers et al., 2017)
		1 mg/kg N + 3 mg/kg I q3w × 4 doses, followed by 3 mg/kg N q2w	40.4% ORR 69.6% OS	61.7% Grade 3/4 TRAEs		
		3 mg/kg N + 1 mg/kg I q3w × 4 doses, followed by 3 mg/kg N q2w	55.2% ORR 80% OS	93% TRAEs 46% Grade 3/4 AEs	NCT02231749 Patients with advanced renal-cell carcinoma with a clear-cell component	(Motzer et al., 2018)
		50 mg sunitinib q1d for 4 weeks	25.5% ORR 72% OS	97% TRAEs 63% Grade 3/4 AEs		
Colorectal cancer	Nivolumab + ipilimumab	3 mg/kg N + 1 mg/kg I q3w × 4 doses, followed by 3 mg/kg N q2w	55% ORR 85% OS	–	CheckMate-142 Patients recurrent CRC or mCRC assessed as dMMR and/or MSI-H per local guidelines	(Overman et al., 2018)

### Pembrolizumab Based Combinational Therapy

#### Melanoma

Standard-dose pembrolizumab given in combination with four doses of reduced-dose ipilimumab followed by standard-dose pembrolizumab has a manageable toxicity profile and provides robust anti-tumor activity in patients with advanced melanoma. These data suggest that standard-dose pembrolizumab plus reduced-dose ipilimumab might be a tolerable, efficacious treatment option for patients with advanced melanoma (Clinical Trials: NCT02089685) (Long et al., 2017).

For melanoma brain metastases patients, Radiosurgery/stereotactic radiotherapy in combination with immunotherapy and targeted agents has been shown to be feasible and well tolerable (Trino et al., 2017).

A phase Ib trial evaluated intratumoral SD-101, a synthetic CpG oligonucleotide that stimulates Toll-like receptor 9 (TLR9), in combination with pembrolizumab in patients with unresectable or metastatic malignant melanoma. Results indicated that the combination of pembrolizumab with intratumoral SD-101 is well tolerated and can induce immune activation at the tumor site. Combining an intratumoral TLR9 innate immune stimulant with PD-1 blockade can potentially increase clinical efficacy with minimal additional toxicity relative to PD-1 blockade alone (Clinical Trials: NCT02521870) (Ribas et al., 2018).

#### NSCLC

Pembrolizumab is a humanized monoclonal antibody against programmed death 1 (PD-1) that has antitumor activity in advanced non-small-cell lung cancer (NSCLC), with increased activity in tumors that express programmed death ligand 1 (PD-L1). In patients with advanced NSCLC and PD-L1 expression on at least 50% of tumor cells, pembrolizumab was associated with significantly longer progression-free and OS and with fewer adverse events than was platinum-based chemotherapy (Clinical Trials: NCT02142738) (Reck et al., 2016).

More recently, pembrolizumab plus chemotherapy was shown to be an effective and tolerable first-line treatment option for patients with advanced non-squamous NSCLC. Cohort G of KEYNOTE-021 (NCT02039674) evaluated the efficacy and safety of pembrolizumab plus pemetrexed-carboplatin (PC) versus PC alone as first-line therapy for advanced nonsquamous NSCLC. At the primary analysis (median follow-up time 10.6 months), pembrolizumab significantly improved ORR and PFS; the hazard ratio (HR) for OS was 0.90 (95% confidence interval [CI]: 0.42‒1.91) (Langer et al., 2016).

The updated analysis indicated that significant improvements in PFS and ORR with pembrolizumab plus PC versus PC alone observed in the primary analysis were maintained, and the HR for OS with a 24-month median follow-up was 0.56, favoring pembrolizumab plus PC (Borghaei et al., 2019).

In patients with previously untreated metastatic nonsquamous NSCLC without EGFR or ALK mutations, the addition of pembrolizumab to standard chemotherapy of pemetrexed and a platinum-based drug resulted in significantly longer OS and PFS than chemotherapy alone (Clinical Trials: NCT02578680) (Gandhi et al., 2018).

In patients with previously untreated metastatic, squamous NSCLC (Clinical Trials: NCT02775435), the addition of pembrolizumab to chemotherapy with carboplatin plus paclitaxel or nab-paclitaxel resulted in significantly longer OS and PFS than chemotherapy alone (Paz-Ares et al., 2018).

Insinga RP et al. describe cost-effectiveness of pembrolizumab plus platinum and pemetrexed chemotherapy in metastatic, non-squamous, NSCLC patients in the US. As a result, the addition of pembrolizumab to chemotherapy is projected to extend life expectancy to a point not previously seen in previously untreated metastatic non-squamous NSCLC. Although ICERs vary by sub-group and comparator, results suggest pembrolizumab + chemotherapy yields ICERs near, or in most cases, well below a 3-times US per capita GDP threshold of $180,000/QALY, and may be a cost-effective first-line treatment for metastatic non-squamous NSCLC patients (Insinga et al., 2018).

#### Renal-Cell Carcinoma

The treatment combination of axitinib plus pembrolizumab is tolerable and shows promising antitumour activity in patients with treatment-naive advanced renal cell carcinoma (Clinical Trials: NCT02133742) (Atkins et al., 2018). In addition, among patients with previously untreated advanced renal-cell carcinoma, treatment with pembrolizumab plus axitinib resulted in significantly longer OS and PFS, as well as a higher ORR, than treatment with sunitinib (Clinical Trials: NCT02853331) (Rini et al., 2019).

#### Advanced Solid Tumors

Purpose Stereotactic body radiotherapy (SBRT) may stimulate innate and adaptive immunity to augment immunotherapy response. Multisite SBRT is an emerging paradigm for treating metastatic disease. Anti-PD-1-treatment outcomes may be improved with lower disease burden. A phase I study to evaluate the safety of pembrolizumab with multisite SBRT in patients with metastatic solid tumors and indicated that multisite SBRT followed by pembrolizumab was well tolerated with acceptable toxicity. Additional studies exploring the clinical benefit and predictive biomarkers of combined multisite SBRT and PD-1-directed immunotherapy are ongoing (Luke et al., 2018).

The phase Ib study (NCT02179918) evaluated the safety, antitumor activity, pharmacokinetics, and pharmacodynamics of utomilumab, a fully human IgG2 mAb agonist of the T-cell costimulatory receptor 4-1BB/CD137 in combination with the humanized, PD-1-blocking IgG4 mAb pembrolizumab in patients with advanced solid tumors. Results showed that patients received combination treatment with no dose-limiting toxicities. Treatment-emergent adverse events were mostly grades 1 to 2, without any treatment-related discontinuations. 26.1% patients had confirmed complete or partial responses (Tolcher et al., 2017).

#### Gastric/Gastroesophageal Junction Cancer

The multicohort, phase II, nonrandomized KEYNOTE-059 study evaluated pembrolizumab ± chemotherapy in advanced gastric/gastroesophageal junction cancer. In detail, in the combination therapy and monotherapy cohorts, 25 and 31 patients were enrolled; median follow-up was 13.8 months (range 1.8–24.1) and 17.5 months (range 1.7–20.7), respectively. In the combination therapy cohort, grade 3/4 treatment-related adverse events occurred in 19 patients (76.0%); none were fatal. In the monotherapy cohort, grade 3–5 treatment-related adverse events occurred in seven patients (22.6%); one death was attributed to a treatment-related adverse event (pneumonitis). The ORR was 60.0% [95% confidence interval (CI), 38.7–78.9] (combination therapy) and 25.8% (95% CI 11.9–44.6) (monotherapy). This study indicated that pembrolizumab demonstrated antitumor activity and was well tolerated as monotherapy and in combination with chemotherapy in patients with previously untreated advanced gastric/gastroesophageal junction adenocarcinoma (Bang et al., 2019).

The details for clinical trials of pembrolizumab based combinational therapy were summarized in **Table 3**.

**Table 3:** Pembrolizumab based combinational therapy.

Cancer type	Treatment	Dose schedule	Efficacy	Adverse rate	Notes	References
Melanoma	Pembrolizumab + ipilimumab	2 mg/kg P + 1 mg/kg I q3w × 4 doses, followed by 2 mg/kg P q3w for up to 2 years	61% ORR 89% OS	45% Grade 3/4 TRAEs	NCT02089685 Patients with advanced melanoma	(Long et al., 2017)
	SD-101 + pembrolizumab	1, 2, 4, or 8 mg SD-101 (Naive to prior anti-PD-1/PD-L1 therapy)	ORR 78%	–	NCT0252189 Patients with unresectable or metastatic malignant melanoma	(Ribas et al., 2018)
		1, 2, 4, or 8 mg SD-101 (Received prior anti-PD-1/PD-L1 therapy)	ORR 15%	–		
	Pembrolizumab ± chemotherapy	200 mg P for four cycles + 5 mg/ml/min (AUC) carboplatin + 500 mg/m^2^ pemetrexed q3w, followed by P for 24 months + pemetrexed maintenance	55% ORR	93% TRAEs	NCT02039674 Patients with chemotherapy-naive, stage IIIB, or IV, non-squamous NSCLC	(Langer et al., 2016)
		Carboplatin + pemetrexed for four cycles, followed by pemetrexed maintenance	29% ORR	90% TRAEs		
	Pembrolizumab ± PC	500 mg/m^2^ pemetrexed + 5 mg/ml/min (AUC) carboplatin q3w for four cycles + 200 mg P q3w for 2 years	56.7% ORR	16.9% TRAEs	NCT02039674 MK-3475-021/KEYNOTE-021 Patients with stage IIIB/IV nonsquamous NSCLC	(Borghaei et al., 2019)
		500 mg/m^2^ pemetrexed + 5 mg/ml/min (AUC) carboplatin q3w for four cycles	30.2% ORR	12.9% TRAEs		
	Pembrolizumab ± Pemetrexed + platinum-based drug	Pemetrexed + platinum-based drug+ 200 mg P q3w for four cycles, followed by P for up to 35 cycles + pemetrexed maintenance	69.2% OS	–	NCT02578680 KEYNOTE-189 Patients with metastatic non-squamous NSCLC	(Gandhi et al., 2018)
		Pemetrexed + platinum-based drug q3w for four cycles, followed by pemetrexed maintenance	49.4% OS			
	Pembrolizumab ± carboplatin + [nab]-paclitaxel	200 mg P (D1) for up to 35 cycles + 6 mg/ml/min (AUC) carboplatin (D1) + 200 mg/m^2^ paclitaxel (D1) or 100 mg/m^2^ nab-paclitaxel (D1, 8, and 15) for the first four cycles	15.9 months mOS	98.2% AEs 69.8% Grade > 3 AEs	NCT02775435 KEYNOTE-407 Patients with untreated metastatic, squamous NSCLC	(Paz-Ares et al., 2018)
		200 mg P (D1) for up to 35 cycles	13.2 months mOS	97.9% AEs 68.2% Grade ≥ 3 AEs		
Renal-cell carcinoma	Pembrolizumab + axitinib	5 mg axitinib q2d + 2 mg/kg P q3w	73% ORR 20.4 months mOS	65% Grade ≥ 3 AEs 54% TRAEs	NCT02133742 Patients with advanced renal cell carcinoma (predominantly clear cell subtype)	(Atkins et al., 2018)
		200 mg P q3w + 5 mg axitinib q2d	59.3% ORR 15.1 months mPFS	75.8% Grade ≥ 3 AEs	NCT02853331 KEYNOTE-426 Patients with untreated advanced clear-cell renal-cell carcinoma	(Rini et al., 2019)
		50 mg sunitinib q1d for the first 4 weeks of each 6-week cycle	35.7% ORR 11.1 months mPFS	70.6% Grade ≥ 3 AEs		
Advanced solid tumors	SBRT + pembrolizumab	SBRT + 200 mg P q3w (within 7 days)	ORR 13.2% 9.6 months mOS 3.1 months mPFS	–	NCT02608385 Patients with metastatic solid tumor previously treated with standard-of-care therapy	(Luke et al., 2018)
	Pembrolizumab + utomilumab	2 mg/kg P q3w + 0.45–5.0 mg/kg utomilumab	26.1% ORR	–	NCT02179918 Patients with advanced/metastatic solid tumor malignancy	(Tolcher et al., 2017)
Gastric/gastroesophageal junction cancer	Pembrolizumab ± chemotherapy	200 mg P for over 30 min infusion (D1) + 80 mg/m^2^ cisplatin (D1) for up to six cycles + 800 mg/m^2^ 5-fluorouracil (D1–5 of each 21-day cycle) for continuous infusion	60.0% ORR	100% TRAEs	NCT02335411 KEYNOTE-059 Patients with recurrent or metastatic G/GEJ adenocarcinoma	(Bang et al., 2019)
		200 mg pembrolizumab for over 30 min infusion (D1 of each 21-day cycle)	25.8% ORR	77.4% TRAEs		

### Atezolizumab Based Combinational Therapy

#### NSCLC and SCLC

Atezolizumab, which restores anticancer immunity, improved OS in patients with previously treated NSCLC and also showed clinical benefit when combined with chemotherapy as first-line treatment of NSCLC. To assess the efficacy and safety of atezolizumab plus chemotherapy versus chemotherapy alone as first-line therapy for non-squamous NSCLC, IMpower130 showed a significant and clinically meaningful improvement in OS and a significant improvement in PFS with atezolizumab plus chemotherapy, than chemotherapy as first-line treatment of patients with stage IV non-squamous NSCLC and no ALK or EGFR mutations. No new safety signals were identified. This study supports the benefit of atezolizumab, in combination with platinum-based chemotherapy, as first-line treatment of metastatic non-small-cell lung cancer (Clinical Trials: NCT02367781) (West et al., 2019).

The phase Ib clinical trial NCT01633970 involved patients with metastatic or locally advanced NSCLC (n = 30) who received 15 mg/kg atezolizumab at 3-week intervals combined with standard chemotherapy (carboplatin + paclitaxel, pemetrexed, or nab-paclitaxel for a total of 4–6 cycles and then maintained with atezolizumab until progression). The ORR was 67% (18 partial responses; two complete responses) (Markham, 2016; Liu et al., 2018).

The addition of atezolizumab to chemotherapy in the first-line treatment of extensive-stage small-cell lung cancer resulted in significantly longer OS and PFS than chemotherapy alone. (Clinical Trials: NCT02763579) (Horn et al., 2018).

#### Breast Cancer

Atezolizumab plus nab-paclitaxel prolonged PFS among patients with metastatic triple-negative breast cancer in both the intention-to-treat population and the PD-L1-positive subgroup. Adverse events were consistent with the known safety profiles of each agent (Clinical Trials: NCT02425891) (Schmid et al., 2018).

In the phase Ib clinical trial NCT01633970, patients diagnosed with triple-negative breast cancer received atezolizumab (800 mg at 2-week intervals) plus nab-paclitaxel (125 mg/m^2^, once a week for 3 weeks in a 4-week treatment course), and five patients were evaluated for efficacy at three-month follow up (four partial responses and one complete response) (Markham, 2016; Liu et al., 2018).

#### Renal-Cell Carcinoma

In the phase Ib clinical trial NCT01633970, patients (n = 12) diagnosed with metastatic renal cell carcinoma received atezolizumab (20 mg/kg) plus bevacizumab (15 mg/kg, at 3-week intervals). At a minimum follow up of 2.1 months, a total of 10 evaluable patients exhibited an ORR of 40%. This study indicated that atezolizumab in combination with bevacizumab enhances antigen-specific T-cell migration in metastatic renal cell carcinoma (Wallin et al., 2016).

The details for clinical trials of atezolizumab based combinational therapy were summarized in **Table 4**.

**Table 4:** Atezolizumab based combinational therapy.

Cancer type	Treatment	Dose schedule	Efficacy	Adverse rate	Notes	References
NSCLC and SCLC	Atezolizumab + Chemotherapy	1200 mg A q3w + 6 mg/ml/min (AUC) carboplatin q3w + 100 mg/m² nab-paclitaxel q1w	18.6 months mOS	24% Serious TRAEs	NCT02367781 Patients with stage IV non-squamous NSCLC	(West et al., 2019)
		6 mg/ml/min (AUC) carboplatin q3w + 100 mg/m² nab-paclitaxel q1w for 4 or 6 21-day cycles, followed by maintenance therapy	13.9 months mOS	13% Serious TRAEs		
	Atezolizumab + platinum-based doublet chemotherapy	15 mg/kg A + 6 mg/ml (AUC) carboplatin q3w + 200 mg/m^2^ paclitaxel q3w	36% ORR 12.9 months mOS	76% Grade≥3 TRAEs	NCT01633970 Patients with stage IIIB/IV NSCLC	(Markham, 2016; Liu et al., 2018)
		15 mg/kg A + 6 mg/ml (AUC) carboplatin q3w + 500 mg/m^2^ pemetrexed q3w	68% ORR 18.9 months mOS	52% Grade≥3 TRAEs		
		15 mg/kg A + 6 mg/ml (AUC) carboplatin q3w + 100 mg/m^2^ nab-paclitaxel q1w	46% ORR 17 months mOS	89% Grade≥3 TRAEs		
	Atezolizumab + Carboplatin and Etoposide	5 mg/ml/min (AUC) carboplatin for four 21-day cycles + 100 mg/m^2^ etoposide (D1-3 of each cycle) + 1200 mg A (D1 of each cycle)	60.2% ORR 12.3 months mOS	56.6% Grade 3/4 AEs	NCT02763579 Patients with extensive-stage SCLC	(Horn et al., 2018)
		5 mg/ml/min (AUC) carboplatin for four 21-day cycles + 100 mg/m^2^ etoposide (D1-3 of each cycle)	64.4% ORR 10.3 months mOS	56.1% Grade 3/4 AEs		
Breast cancer	Atezolizumab ± nab-paclitaxel	840 mg A (D1 and 15) + 100 mg/m^2^ nab-paclitaxel (D1, 8, and 15) for 28-day cycle	56.0% ORR 21.3 months mOS	48.7% Grade 3/4 AEs	NCT02425891 Patients with metastatic TNBC	(Schmid et al., 2018)
		100 mg/m^2^ nab-paclitaxel (D1, 8, and 15) for 28-day cycle	45.9% ORR 17.6 months mOS	42.2% Grade 3/4 AEs		

### Durvalumab Based Combinational Therapy

#### NSCLC

Clinical Trials NCT02000947 assess durvalumab plus tremelimumab in patients with advanced squamous or non-squamous NSCLC. Durvalumab 20 mg/kg every 4 weeks plus tremelimumab 1 mg/kg showed a manageable tolerability profile, with antitumor activity irrespective of PD-L1 status (Antonia et al., 2016).

Clinical trial NCT02088112 evaluated the combinational therapy of durvalumab (10 mg/kg intravenously Q2W) plus gefitinib (250 mg once daily) in TKI-naive patients harboring sensitizing EGFR mutations associated with advanced NSCLC (Gibbons et al., 2016). Approximately 10 patients were assigned to group 1 and given durvalumab + gefitinib, whereas the other 10 patients of group 2 were administered gefitinib monotherapy for the first 4 weeks, followed by gefitinib plus durvalumab (Gibbons et al., 2016). The results observed grade 3–4 adverse effects, and the treatment was discontinued in four patients (all included in arm 2). Observed partial response (PR) or complete response (CR) was 77.8% or 80%, respectively, in patients belonging to group 1 and 2 (Gibbons et al., 2016).

#### Women's Cancers

A study of the PD-L1 inhibitor, durvalumab, in combination with a PARP inhibitor, olaparib, and a VEGFR1-3 inhibitor, cediranib, in recurrent women's cancers with biomarker analyses were conducted and results showed that the recommended phase 2 dose (RP2D) is tolerable and has preliminary activity in recurrent women's cancers (Lee J.M. et al., 2017; Zimmer et al., 2019).

A pilot study of durvalumab and tremelimumab and immunogenomic dynamics in metastatic breast cancer showed that responses are low in unselected metastatic breast cancer, however, higher rates of clinical benefit were observed in triple negative breast cancer (TNBC). This study suggested that immunogenomic dynamics may help identify phenotypes most likely to respond to immunotherapy (Santa-Maria et al., 2018).

In the NCT02484404 phase I trial, durvalumab plus olaparib resulted in higher clinical activity in patients diagnosed with triple-negative breast cancer or ovarian cancer in the absence of germline BRCA mutations (Lee J.M. et al., 2017).

In NCT02291055 phase I/II trial, the combinatorial treatment of durvalumab and axalimogene filolisbac were determined to be efficacious in previously treated patients who were diagnosed with HPV-associated cervical cancer (recurrent/metastatic) (Syed, 2017).

#### Prostate Cancer

In metastatic castration-resistant prostate cancer, durvalumab plus olaparib has acceptable toxicity, and the combination demonstrates efficacy, particularly in men with DNA damage repair (DDR) abnormalities (Karzai et al., 2018).

#### Lymphoma

The phase 1b/2, multicenter, open-label study evaluated ibrutinib plus durvalumab in relapsed/refractory follicular lymphoma (FL) or diffuse large B-cell lymphoma (DLBCL). In FL, GCB DLBCL, and non-GCB DLBCL, ibrutinib plus durvalumab demonstrated similar activity to single-agent ibrutinib with the added toxicity of the PD-L1 blockade; the combination resulted in a safety profile generally consistent with those known for each individual agent (Herrera et al., 2020).

#### Melanoma

In the NCT02027961 phase I/II trial, durvalumab + darafenib + trametinib was administered to unresectable patients with wild-type metastatic or BRAF-mutant melanoma (Syed, 2017).

#### Solid Tumors

In the NCT02141347 phase I trial, the combination durvalumab plus tremelimumab resulted in early effects in Japanese patients diagnosed with advanced solid tumors (Syed, 2017).

The details for clinical trials of durvalumab based combinational therapy were summarized in **Table 5**.

**Table 5:** Durvalumab based combinational therapy.

Cancer type	Treatment	Dose schedule	Efficacy	Adverse rate	Notes	References
NSCLC	Durvalumab + tremelimumab	D q4w × 13 doses + T q4w for 6 doses, followed by T q12w × 3 doses Escalation dose of D: 3, 10, 15, 20 mg/kg Escalation dose of T: 1, 3, 10 mg/kg	17% ORR	36% TRAEs	NCT02000947 Patients with locally advanced or metastatic NSCLC, immunotherapy-naïve	(Antonia et al., 2016)
	Durvalumab + gefitinib	10 mg/kg D q2w + 250 mg gefitinib q1d	ORR 77.8%	100% TRAEs	NCT02088112 Patients harboring sensitizing EGFR mutations associated with advanced NSCLC, TKI-naive	(Gibbons et al., 2016)
		250 mg gefitinib q1d for 4 weeks, followed by 10 mg/kg D q2w + 250 mg gefitinib q1d	ORR 80%	100% TRAEs		
Women's cancers	Durvalumab + olaparib	10 mg/kg D q2w or 1,500 mg D q4w + olaparib Escalation dose of olaparib: 200, 300 mg	17% ORR	–	NCT02484404 Patients with TNBC or ovarian cancer	(Lee J.M. et al., 2017)
		10 mg/kg D q2w or 1,500 mg D q4w + cediranib Escalations dose of cediranib: 20, 30 mg	50% ORR	–		
Lymphoma	Durvalumab + ibrutinib	560 mg ibrutinib q1d + 10 mg/kg D q2w for 28-day cycles	25% ORR	20% TRAEs	NCT02401048 Patients with relapsed/refractory DLBCL or FL	(Herrera et al., 2020)

### Avelumab Based Combinational Therapy

#### Preclinical Study

NHS-muIL12 and avelumab combination therapy enhanced antitumor efficacy relative to either monotherapy in two tumor models-BALB/c mice bearing orthotopic EMT-6 mammary tumors and μMt-mice bearing subcutaneous MC38 tumors. Most EMT-6 tumor-bearing mice treated with combination therapy had complete tumor regression. Combination therapy also induced the generation of tumor-specific immune memory, as demonstrated by protection against tumor rechallenge and induction of effector and memory T cells. Combination therapy enhanced cytotoxic NK and CD8^+^ T-cell proliferation and T-bet expression, whereas NHS-muIL12 monotherapy induced CD8^+^ T-cell infiltration into the tumor. Combination therapy also enhanced plasma cytokine levels and stimulated expression of a greater number of innate and adaptive immune genes, compared with either monotherapy. These data indicate that combination therapy with NHS-muIL12 and avelumab increased antitumor efficacy in preclinical models, and suggest that combining NHS-IL12 and avelumab may be a promising approach to treating patients with solid tumors (Xu et al., 2017).

#### Renal-Cell Carcinoma

In a single-group, phase 1b trial, avelumab plus axitinib resulted in objective responses in patients with advanced renal-cell carcinoma (Choueiri et al., 2018).

The next phase 3 trial involving previously untreated patients with advanced renal-cell carcinoma compared avelumab plus axitinib with the standard-of-care sunitinib. PFS was significantly longer with avelumab plus axitinib than with sunitinib among patients who received these agents as first-line treatment for advanced renal-cell carcinoma (Clinical Trials: NCT02684006) (Motzer et al., 2019).

#### Head and Neck Cancer

The JAVELIN Head and Neck 100 study is a multinational, Phase III, double-blind, placebo-controlled, randomized clinical trial assessing the efficacy of avelumab, a PD-L1 inhibitor, in combination with CRT compared with placebo in combination with CRT for high-risk HNSCC (Trial registration: Javelin Head and Neck 100; NCT 02952586) (Yu and Lee, 2019).

### Cemiplimab Based Combinational Therapy

#### Preclinical Study

In an engineered T cell/antigen-presenting cell (APC) bioassay, REGN3767 alone, or in combination with cemiplimab (REGN2810, human anti-PD-1 Ab), blocked inhibitory signaling to T cells mediated by hLAG-3/MHCII in the presence of PD-1/PD-L1. To test the *in vivo* activity of REGN3767 alone or in combination with cemiplimab, human PD-1×LAG-3 knock-in mice were generated, in which the extracellular domains of mouse Pdcd1 and Lag3 were replaced with their human counterparts. In these humanized mice, treatment with cemiplimab and REGN3767 showed increased efficacy in a mouse tumor model and enhanced the secretion of proinflammatory cytokines by tumor-specific T cells. The favorable pharmacokinetics and toxicology of REGN3767 in non-human primates, together with enhancement of antitumor efficacy of anti-PD-1 Ab in preclinical tumor models, supports its clinical development (Burova et al., 2019).

### Toripalimab Based Combinational Therapy

A single-center, phase IB trial (NCT03086174) evaluated the safety and preliminary efficacy of toripalimab combined with the VEGF receptor inhibitor axitinib in patients with advanced melanoma, including chemotherapy-naive mucosal melanomas). 33 patients were enrolled to receive 1 or 3 mg/kg toripalimab every 2 weeks, in combination with 5 mg axitinib twice a day, in a dose-escalation and cohort-expansion study. The results showed no dose-limiting toxicities observed, while 97% patients experienced treatment-related adverse events (TRAEs). The most common TRAEs were mild, while grade 3 or greater TRAEs occurred in 39.4% of patients. Among patients with chemotherapy-naive mucosal melanoma, 48.3% patients achieved objective response, and the median PFS was 7.5 months. Although the combination therapy was tolerable and showed promising antitumor activity, due to patients enrolled in this study were all Asian, these results must be validated in a randomized phase III trial that includes a non-Asian population (Sheng et al., 2019).

### Camrelizumab Based Combinational Therapy

The first-line standard of care for patients with recurrent or metastatic nasopharyngeal carcinoma are platinum-based doublet chemotherapy regimens, specially gemcitabine combined with cisplatin. Two single-arm, phase 1 trials (NCT02721589 and NCT03121716) were designed to evaluate the safety and preliminary anti-tumor activity of camrelizumab in combination with gemcitabine plus cisplatin for patients with recurrent or metastatic nasopharyngeal carcinoma. Camrelizumab combined with first-line standard therapy exhibited a manageable toxicity profile and promising preliminary anti-tumor activity for this disease in treatment-naive patients (Fang et al., 2018).

### Tislelizumab Based Combinational Therapy

A multicentre, open-label, phase 1a/b study (NCT02660034) was designed to investigate the safety and anti-tumor effects of pamiparib, PARP 1/2 inhibitor, in combination with tislelizumab. Forty-nine patients with advanced solid tumors were enrolled to determine the optimum doses for further evaluation. The recommended phase 2 dose was determined as tislelizumab 200 mg every 3 weeks in combination with pamiparib 40 mg twice daily. Pamiparib plus tislelizumab exhibited generally well tolerance and were associated with anti-tumor responses and clinical benefit in patients with advanced solid tumors, supporting further investigation of the combined therapy (Friedlander et al., 2019).

## Toxicity and Side Effects Caused by PD-1/PD-L1-Based Monotherapy or Combination Therapy

Similar to any other drug, checkpoint inhibitors provide benefits as well as risks. Generally speaking, side effects of PD-1 inhibitors are less common than those of CTLA-4 inhibitors. The spectrum of side effects caused by PD-1/PD-L1 inhibitors includes gastrointestinal, hepatic, dermatologic, and endocrine events (Naidoo et al., 2016; Davis et al., 2017). It is usually recommended that patients with grade 2 toxicity should refrain from receiving checkpoint inhibitors transiently. For patients exhibiting grade 3 or higher adverse effects, treatment should be terminated and systemic corticosteroids should be given (1 to 2 mg/kg or equivalent) daily (Naidoo et al., 2016; Davis et al., 2017).

Data from mouse gene knockout studies indicated that blocking the PD-1/PD-L1 pathway results in relatively low incidence of autoimmune reactions that can be managed with immune suppression or supportive care. Toxicological studies involving monkeys indicated gastrointestinal toxicity may reach grades 3 to 4 after application of nivolumab and ipilimumab (Sznol, 2014). Toxicities due to combinational treatment of nivolumab + ipilimumab are similar to that generated using ipilimumab alone. In return for high rates of activity and efficacy, high rates of reversible autoimmune adverse events of grade 3 to 4 caused by combination regimens could be tolerated if toxicities are reversible with acceptable morbidity (Sznol, 2014). Combining anti-PD-1/PD-L1 inhibitors with chemotherapeutic agents was reported in quite a few clinical trials. There was a single-center phase Ib study investigating the tolerability and safety of nivolumab combined with standard chemotherapy in patients with NSCLC. Skin toxicities and hepatic toxicities were more frequently than chemotherapy or nivolumab alone, they were mild and intervention with systemic corticosteroids was not needed. Only two patients with interstitial lung disease were resolved by systemic corticosteroids, which happened in two patients several months after the start of treatment. It suggests that combination therapy with nivolumab and standard chemotherapy strengthens the anti-tumor activity of each monotherapy (Kanda et al., 2016).

Thyroid disorders are one of the most common adverse events caused by anti-PD-1 monotherapy or combinatorial therapy of anti-CTLA-4 plus anti-PD-1 (Lee H. et al., 2017). Studies comparing the prevalence of drug-related thyroid disorders due to monotherapy or combination therapy have been performed. The dynamic evolution of thyroid disorders has also been assessed in 45 patients who received anti-PD-1 monotherapy or anti-CTLA-4/anti-PD-1 combinatorial therapy. Results indicate that thyrotoxicosis or hypothyroidism are the initial form of thyroid disorders (Lee H. et al., 2017). Thyrotoxicosis occurs in most of the treated patients, with a prevalence of 93% for combination therapy and 56% for monotherapy. Additionally, the onset pattern of the thyroid disorder differs significantly between these two groups (p = 0.01). Subsequently, 76% and 90% of thyrotoxicosis shifted into hypothyroidism in patients of combination and monotherapy groups, respectively (Lee H. et al., 2017). The median time for onset of thyrotoxicosis and hypothyroidism was 31 and 68 days after first treatment, and 21 and 63 days for monotherapy groups and combination therapy, respectively. The median time was 42 days for the transition from thyrotoxicosis to hypothyroidism in both groups (Lee et al., 2017).

The most common side effects include immune-related and were observed in about 60% of patients enrolled in phase II and III studies. These side effects were mainly low grade and the majority involved skin conditions such as pruritus and rash or GI conditions, including diarrhea and colitis (Weinstock et al., 2017).

## Prospects

Immunotherapy based on PD-1/PD-L1 has revealed its efficacy in melanoma, NSCLC, gastric cancer, as well as head and neck cancer. The frequency of side effects of PD-1/PD-L1 therapy due to immune suppression is relatively lower than using traditional cancer therapy and are better tolerated. However, due to the immunomodulating nature of the mAbs, the measurement of the biological activities (release or stability test) made a great problem in quality control laboratories (Wang et al., 2017). As therapeutic antibodies, the limited half-life and multiple-dosages-caused immunogenicity, which might induce over-activity of immune system, were inevitably emerged, some small-molecule immune checkpoint inhibitors to avoid these shortcomings are under developing (Lee et al., 2016; Magiera-Mularz et al., 2017; Li and Tian, 2019). The above factors made these drugs a high cost for biopharmaceutical industrials, which is not conducive to benefit more patients (Kandolf Sekulovic et al., 2017; Ward et al., 2017).

Despite some disadvantages, checkpoint inhibitors possess a great prospect. The recent findings suggest that PD-1/PD-L1 inhibitors may be combined with other immunotherapies or traditional treatments to enhance efficacy relative to that using PD-1/PD-L1 therapy alone, which always exhibit higher response rates, reducing adverse reaction and drug resistance (Li J. et al., 2019; Zhang et al., 2019; Li et al., 2020; Shao et al., 2020; Sonpavde et al., 2020; Wan et al., 2020; Weiss et al., 2020; Zhang et al., 2020). Some researchers have shown the prospects of anti-PD-L1 and anti-CTLA-4 combination therapy, which revealed PD-L1:CD80 (CTLA-4 ligand) cis-heterodimerization inhibited both PD-L1:PD-1 and CD80:CTLA-4 interactions. Therefore, exploration of the efficacy and mechanism of co-blockade of PD-L1 and CTLA-4 is promising (Sugiura et al., 2019; Zhao et al., 2019). The emerging nanovaccine was reported to profoundly potentiate the immunogenicity of the neoantigen, enhancing responsiveness (Ni et al., 2020). Furthermore, some studies reveal that angiotensin-converting enzyme 2 (ACE2) expression is increased after interleukin (IL)-1β treatment (Clarke et al., 2014), blockade of IL-1β synergized with blockade of PD-1 can inhibit tumor growth (Tian et al., 2020). This correlation can provide new ideas for anti-PD-1/PD-L1 therapy (Sui et al., 2014). Above all, the combination therapy using PD-1/PD-L1 may pave the way for a new era for cancer immunotherapy.

## Author Contributions

L-WF conceived the review. J-YZ and Y-YY searched the literature and drafted the manuscript. J-JL revised literature. RA edited the manuscript. All authors approved the final version of the manuscript.

## Funding

This work was supported by National Natural Science Foundation of China (81773888, U1903126 and 81902152), Natural Science Foundation of Guangdong Province (2020A1515010605), Fund of Guangzhou Science and Technology Program (201707010048), Open Funds of State Key Laboratory of Oncology in South China (HN2018-06), the Fund of Shanxi Province Higher Education Technology Innovation Project (2019L0753).

## Conflict of Interest

The authors declare that the research was conducted in the absence of any commercial or financial relationships that could be construed as a potential conflict of interest.
